# Bioinformatics strategies for taxonomy independent binning and visualization of sequences in shotgun metagenomics

**DOI:** 10.1016/j.csbj.2016.11.005

**Published:** 2016-12-05

**Authors:** Karel Sedlar, Kristyna Kupkova, Ivo Provaznik

**Affiliations:** Department of Biomedical Engineering, Brno University of Technology, Technicka 12, Brno, Czech Republic

**Keywords:** Metagenomics, Taxonomy independent, Sequence binning, Genomic signature, Abundance, Visualization

## Abstract

One of main steps in a study of microbial communities is resolving their composition, diversity and function. In the past, these issues were mostly addressed by the use of amplicon sequencing of a target gene because of reasonable price and easier computational postprocessing of the bioinformatic data. With the advancement of sequencing techniques, the main focus shifted to the whole metagenome shotgun sequencing, which allows much more detailed analysis of the metagenomic data, including reconstruction of novel microbial genomes and to gain knowledge about genetic potential and metabolic capacities of whole environments. On the other hand, the output of whole metagenomic shotgun sequencing is mixture of short DNA fragments belonging to various genomes, therefore this approach requires more sophisticated computational algorithms for clustering of related sequences, commonly referred to as sequence binning. There are currently two types of binning methods: taxonomy dependent and taxonomy independent. The first type classifies the DNA fragments by performing a standard homology inference against a reference database, while the latter performs the reference-free binning by applying clustering techniques on features extracted from the sequences. In this review, we describe the strategies within the second approach. Although these strategies do not require prior knowledge, they have higher demands on the length of sequences. Besides their basic principle, an overview of particular methods and tools is provided. Furthermore, the review covers the utilization of the methods in context with the length of sequences and discusses the needs for metagenomic data preprocessing in form of initial assembly prior to binning.

## 1 Introduction

Direct sequencing of genomic material from an environment, commonly referred to as metagenomics, helped to provide a full insight into entire microbial communities that could not have been studied before for a majority of the organisms are uncultivable [1]. Nowadays, there are thousands of metagenomic projects compared with only few studies published in early 2000s [2], [3]. The aim of these projects is to explore microbiologically diverse environments such as soil [4], marine water [5], gut or other niches of human [6] or other higher eukaryotes. Each of these habitats is characterized by specific taxonomic composition of particular genomes and every genome by its specific composition of genes, forming the resulting metabolism [7]. Metagenomic research can therefore infer medically or industrially important knowledge by revealing hitherto undescribed genes responsible for antibiotic resistance or enzyme synthesis [8]. To further underline the importance of metagenomic research, it was estimated that the microbial cell population of human genome outnumbers the number of human own cells by 10 fold [9], leading to conclusion that the influence of microbiota on human health is far greater than it was expected. One of the basic problems in metagenomic studies remains in taxonomic classification of the sequences within the sample. This task is a challenging one since the volume of metagenomic data is rather large and therefore imposes high demands on bioinformatic tools for fast and effective data processing. Furthermore, there is no prior knowledge about the species richness of the sample which makes the classification process even more challenging. In metagenomics, the assignment of genomic fragments to the corresponding taxonomic group, e.g. species, genera or higher taxonomic groups, is commonly referred to as “binning” as each of the sequences is placed into an imaginary bin representing ideally only fragments belonging to this group. The outcome of the binning process can then be used not only for taxonomic diversity assessment, but also for facilitation of genome assembly, evaluation of gene association with different taxonomic groups or as the basis for following metatranscriptomics or metabolomics analyses leading to revelation of novel interactions [10], [11], [12], [13].

With regards to sequencing strategy, there are two main approaches to study microbiomes. The first strategy is based on amplicon sequencing of a target gene in the metagenome, while the second strategy uses the whole metagenome shotgun (WMS) sequencing [14]. Using specific primers, only phylogenetic marker genes or their parts are sequenced in the first approach. 16S rRNA [15] and internal transcribed spacer (ITS) [16] regions are the most commonly used for prokaryotic and fungal species respectively. Unfortunately, no additional information apart from species richness and abundance can be determined for all sequences represent the same genomic region. Nevertheless, thanks to comprehensive databases of marker genes, the binning process is relatively easy and reliable [17], which makes the amplicon sequencing data analysis a standard approach for the diversity investigation of metagenomes [18] and new techniques for community detection and visualization of microbiomes are still being developed [19]. The solution to the loss of information about metagenome is offered by utilization of the second approach using WMS sequencing. This strategy provides a deep insight into a metagenome, as every sequence represents a random part, including unknown genes, of a genome occurring in the metagenome [20]. On the other hand, the preprocessing of the WMS data can be a challenging task not only for much larger volume of data being processed, but also due to the lack of the reference whole genomic sequences within the available databases. These challenges make the binning process especially difficult. However, a competitive research in bioinformatics strives to solve these issues and therefore a majority of the newly developed algorithms is focused on WMS data processing.

The output of whole metagenome sequencing is formed by genomic fragments, about which there is no taxonomical information available. These fragments can be analogously associated with puzzle pieces belonging to different puzzle sets. Moreover, their length can differ depending on the used sequencing technology [21]. Current assembling technologies are still unable to assemble the short fragments into a whole genome sequences, and often fail at level of contigs [22]; therefore binning process, where fragments are divided into species or strain-level clusters, is essential for better reconstruction of novel microbial genomes to gain knowledge about genetic potential of whole metagenome. There are two different groups of strategies for WMS data binning. The taxonomy dependent, also referred to as supervised, methods rely on comparison of sequences against reference databases. Comparison can be performed (i) on the sequence level using aligning algorithms like BLAST [23], BLAT [24], Bowtie [25], BWA [26], (ii) on the model level of a known phylogenetic origin using Hidden Markov Models (HMM) and specific database such as Pfam [27] or (iii) on a sequence composition level using GC content, oligonucleotide patterns [28], etc. Although there is a wide range of such techniques, they suffer from two major issues. Firstly, the comparison part of the algorithm is time consuming; this especially applies to the aligning methods. Secondly, the reference databases containing whole genome sequences are far from complete. According to estimations the whole prokaryotes group consists of almost 10^8^ separate genospecies [8] and while there is more than 3 million of 16S rRNA genes already sequenced, only around 6000 complete genomes are available up to date [29], [30]. Therefore, a great number of sequences can end up either unassigned or as false positive assignments. The accuracy prediction of the methods then relies on the required taxonomy level of assignment, where the probability of assignment to the correct group rises with the increasing taxonomical level. The second group of methods overcomes both of the mentioned disadvantages by using taxonomy independent also called unsupervised approach. These techniques are based on extraction of parameters specific for given taxon out of the raw sequencing reads or preassembled contigs. The obtained parameters are then directly compared and binned by use of suitable machine learning algorithms, without the need for any reference database.

Although an overall summary of all strategies for metagenomic shotgun data binning, as well as validation strategies for these techniques is summarized in review by Mande et al. [31], the list of described taxonomy independent strategies contains only five techniques. While in taxonomy dependent strategy research authors mainly work on improving the current techniques, the taxonomy independent strategy, which became the main focus of interest and has undergone great development especially over the last five years, expanded by a lot of new techniques. In this mini review, we update the list of taxonomy independent strategies by recently published techniques and provide their further division and description. On top of that, the future trends in the field are briefly discussed at the end of this review.

## 2 Taxonomy independent binning algorithms

**Fig. 1:**
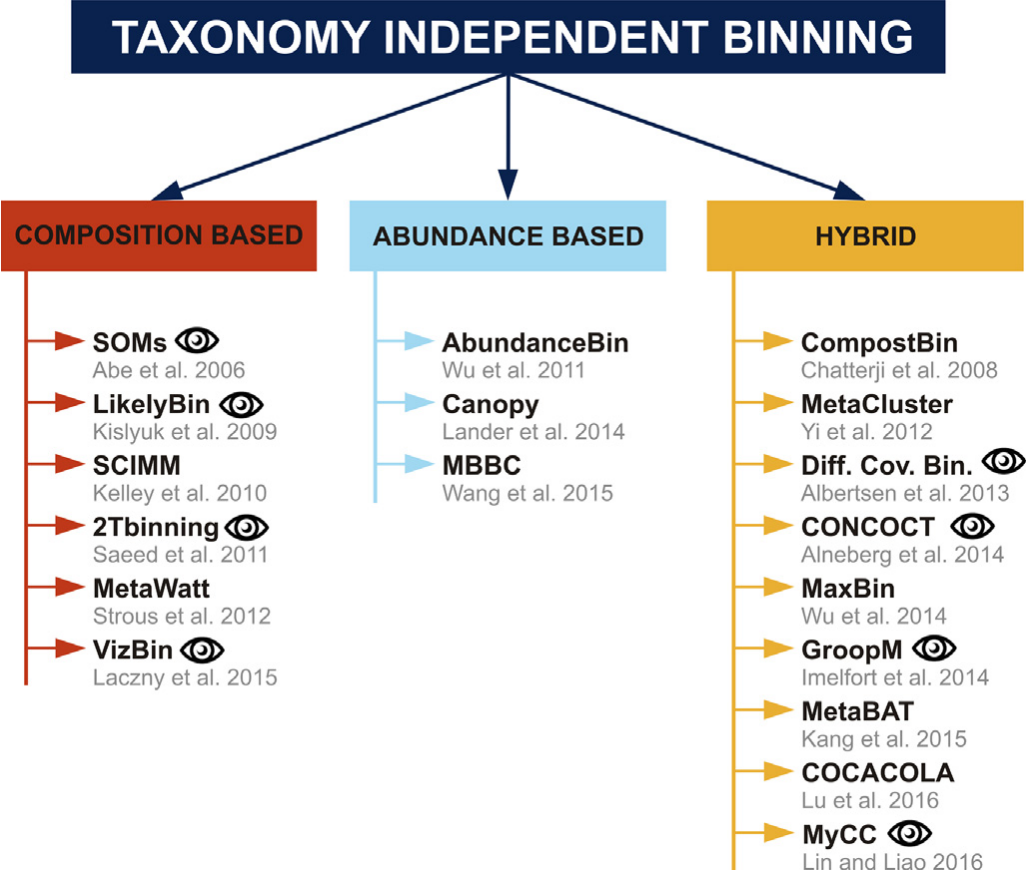
Schematic distribution of current taxonomy independent binning methods into three categories; the eye symbol highlights the methods that enable visualization of datasets.

Rapid development of taxonomy independent strategies for metagenomic data binning brings a wide range of new techniques, utilizing various machine learning, clustering and visualization algorithms. While these techniques usually differ in algorithm they use for binning, they share the strategy for extraction of features on which the binning is performed. There are two basic types of features used for classification, namely features based on sequence composition, and features based on contig coverage reflecting abundance of given taxa in a microbial sample. Considering these parameters, the existing techniques can be divided into three categories, as shown in Fig. 1, specifically: (i) sequence composition based methods, (ii) abundance based methods, and (iii) hybrid methods, which combine both, information regarding the sequence composition as well as taxa abundance.

### 2.1 Sequence composition based binning

**Fig. 2:**
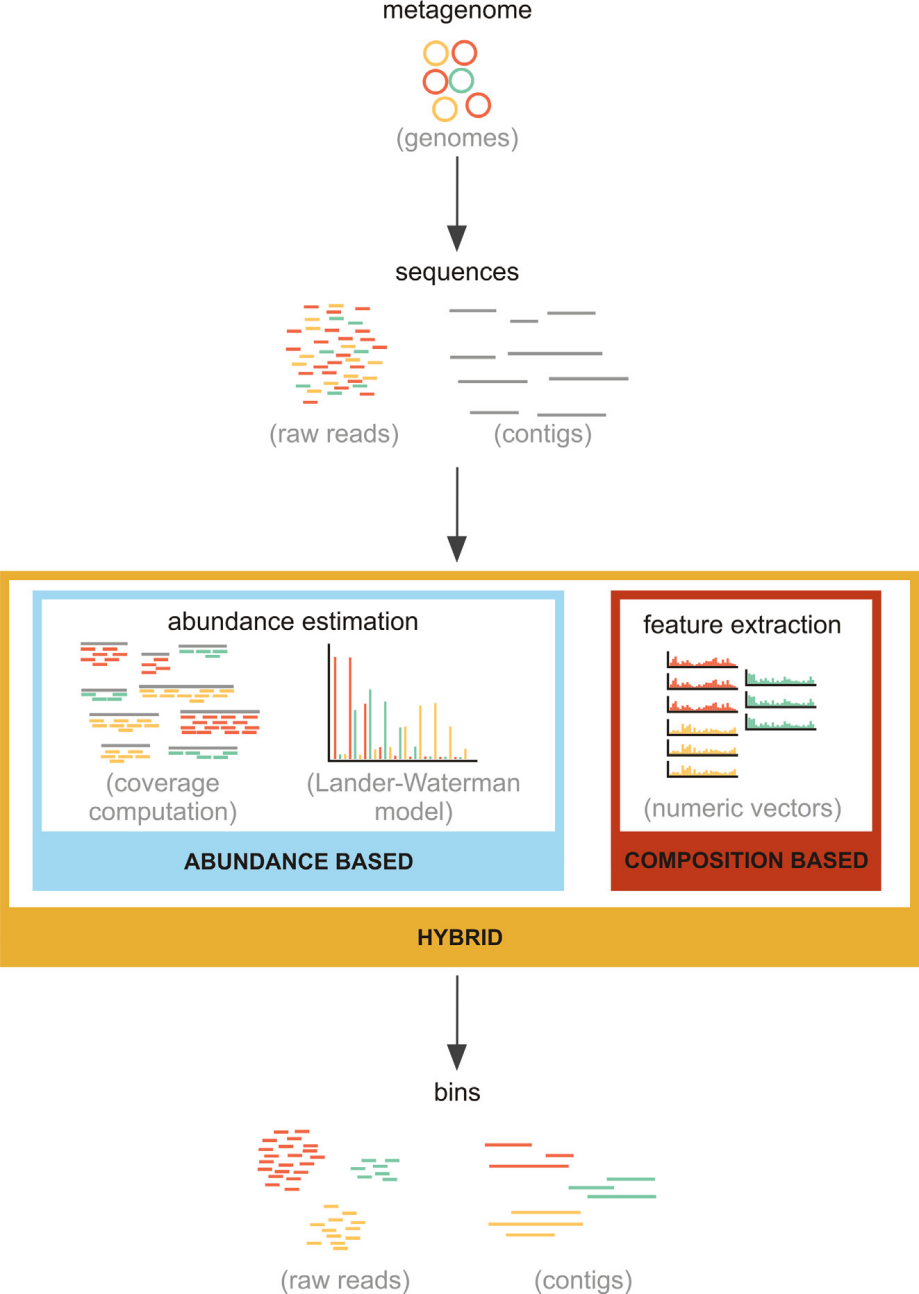
Workflow of taxonomy independent binning strategies.

The core idea of the methods in this section is based on an assumption that the genome composition is unique for each taxon, and therefore it is possible to bin the sequences purely by comparing their content. Since sequence composition is character based, it is essential to first transform it to a suitable numerical feature vector. The most commonly used features are so-called genomic signatures, which are normalized frequencies of *k*-mers of a particular size [32]. Typically, *k* is set to four, which results in high dimensional Euclidean space with 4^4^ = 256 dimensions formed by frequencies of particular words four characters in length {AAAA, AAAC,..., TTTT}. Various strategies can reduce the dimensionality of the vector by different methods, for example to 136 dimensions, when accounting for reversed complements and palindromes [33]. Another parameter that is possible to use for sequence comparison is the guanine-cytosine (GC) content, since studies confirmed difference of GC content among unrelated populations [34]. The common workflow of binning strategies is shown in Fig. 2.

TETRA [35], a tool for statistical analysis and comparison of sequences based on tetranucleotide pattern frequencies, can be considered as a predecessor of modern binning methods. The computing capacity of the tool, however, does not meet the needs of current metagenomics and the tool is no longer available. One example of the current methods using composition based binning is LikelyBin [36], which utilizes the Markov Chain Monte Carlo approach for binning sequences based on *k*-mers of lengths between *k* = 2 and *k* = 5. Despite the fact that the method is fully automatic, its use is limited only on low complexity metagenomes (2–10 species), where the method reaches high accuracy given sufficient genomic divergence. Better result in terms of precision and accuracy were reached by use of the SCIMM [37] technique, which uses interpolated Markov models (IMM) on initial clusters for production of higher quality bins. Unfortunately, initial bins need to be formed before the application of IMM on the data. This can be done either by k-means clustering, which needs a predicted number of clusters as an input, or by running another binning algorithm, e.g. LikelyBin [36] or CompostBin [38]. Although SCIMM can improve the quality of clusters, the final results are highly dependent on this initial step. Also use of SCIMM is limited to lower complexity metagenomes, as both recall, and especially precision values are lower with increasing number of genomes presented within a sample.

Complex microbial samples can be analyzed by use of different forms of self-organizing maps (SOMs) [39], [40], [41], [42], [43]. A SOM is an artificial neural network proposed by Kohonen (1990) [44] for data clustering. Its properties are making it an ideal tool for clustering and visualization of high-dimensional data like genomic signatures by mapping them on a two-dimensional map. One form of SOM is batch-learning SOM (BLSOM) specifically modified for genome informatics to make the learning process and resulting map independent of the order of data input [41], [42]. In order to lower computational demands of BLSOM, a novel method Self-Compressing BLSOM (SC-BLSOM) was invented, which rapidly fastens the clustering process [43]. Although SOM can be an effective tool for cluster analysis, it also has its drawbacks. Firstly, the contour definition and therefore the final clustering can be hard task that significantly affects the results of taxonomic profiling. Secondly, the kernel transformation suffers from quadratic time complexity therefore it is time consuming. A solution to the second addressed problem is offered by VizBin [45] which also reduces the high-dimensional *k*-mers into two-dimensional space by use of the Barnes-Hut Stochastic Neighbor Embedding (BH-SNE) algorithm with time complexity only *O*(*n*log*n*) compared to *O*(*n*^*2*^) of SOM [46], [47]. One of the major advantages of VizBin is that it provides rather distinctly bounded clusters in satisfactory time. On the other hand, the final binning is not automatic and the results are therefore purely dependent on human assessment which can be especially problematic with high-complexity metagenomic data.

Several parameters were combined in 2Tbinning [48], these include GC content, oligonucleotide frequency derived error gradient (OFDEG) [49] and tetramer frequency. 2Tbinning stands for 2-tier binning, as in the first tier sequences are separated into preliminary groups based on GC content and OFDEG parameter, and in the second tier, these groups are then separately divided into finer bins utilizing *k*-mer frequencies. Also MetaWatt [50] is a tool that bins sequences in two steps, where sequences are firstly separated into clusters regarding to an empirical relationship between the mean and standard deviation of tetramer frequencies. The optimal bins are then selected by an expert and used for creation of IMMs, which are then used for improvement of the binning results obtained in the first step, similarly to SCIMM. However, compared with SCIMM, which uses fully automatically defined bins for IMM modeling, MetaWatt requires human input.

### 2.2 Abundance based binning

One of the problems with the composition based methods is the binning of species with low abundance, as sequences belonging to these species form smaller indistinct clusters, which can then be easily misclassified as part of a larger bin belonging to highly abundant species. This issue can be solved by use of abundance based binning methods, which can be further subdivided into methods for working with one sample (AbundanceBin [51], MBBC [52]), and methods working with series of metagenomic samples (Canopy [53]). The key idea of the first group is that the distribution of sequenced reads follows the Lander-Waterman model, where coverage of each nucleotide can be computed by the application of the Poisson distribution [54]. The workflow of these methods is therefore somewhat similar to the composition based binning techniques, with the main difference in cluster formation being defined by *k*-mer abundance (content) instead of their similarity (composition). The second group of methods is based on the assumption that coverage profiles of contigs from the same genomes should be highly correlated across multiple samples. The necessary step lies in de novo assembly of raw reads into contigs, as shown in the schematic workflow in Fig. 2.

The second problem with composition based methods is that they usually provide reasonably accurate results only when longer sequences are used (e.g. 800 bp). AbundanceBin, the one-sample abundance based method, gives solution to this issue and can work accurately even with sequence reads that are only 75 bp long. The technique extracts *l*-tuples (*l* was experimentally estimated to 20) from all reads and then by use of the Expectation–Maximization (EM) algorithm, finds the parameters for the Poisson distributions, which reflect the relative abundance levels of the species. Since AbundanceBin uses a recursive binning approach for bin number estimation, there is no need for human input, which makes the method fully automatic. A user can still possibly change the initial conditions for the EM algorithm for the initial estimation of abundance levels and genome sizes, which are determined empirically for default mode. A similar pipeline to AbundanceBin is introduced in MBBC, where the initial binning is also performed by finding parameters for Poisson distributions by the EM algorithm; however, the outcome is then used for training Markov models, based on which the preliminary bins are refined. Although both of the methods work well even on very short sequences (e.g. 75 bp), the setting of initial conditions can be crucial for the outcome. Moreover, in MBBC, the user is required to enter a large number as an estimation of number of bins. While the number is then optimized, it is still unclear, what can be considered large for different samples, thus testing an optimal setting can be time consuming.

In order to overcome resolution limitations, in form of inability to separate closely related organisms, abundance can be computed and compared across many samples. Such resolution enhancement is introduced in Canopy, which clusters sequences based on gene abundance profiles across many samples. Since the method works only with gene regions, it requires use of MOCAT software package [55] for gene prediction. The canopy-based clustering is then performed simply by searching for genes within a predefined distance from a randomly picked gene, which has not been clustered yet. Bins are then further edited based on the gene content. The method therefore does not require predefined number of clusters or any other human input, making it fully automatic.

### 2.3 Hybrid binning

The hybrid binning methods combine the two aforementioned strategies into a compact technique. It has been previously proven that by combination of information about the sequence composition and coverage, which reflects the species abundance, one can extract more information about metagenomics data, which eventually leads to more accurate binning results [59].

The very first hybrid method was CompostBin [38]. Unlike the majority of sequence composition based methods requiring prior assembly, which can possibly lead to the formation of defective sequences, referred to as chimeric contigs; the workflow of CompostBin is designed in such way that the method can be applied directly onto raw reads. Initial extraction of hexamer frequencies is followed by principle component analysis (PCA) for dimensionality reduction, which is, however, weighted by an inverted value of sequence coverage. By applying the weights, the between species variance is not overwhelmed by within species variance of the more abundant species. Bins are then formed by the application of fully automatic recursive division algorithm on the data in the final lower-dimensional space; thus, no prior knowledge is required. Another method that uses PCA for dimensionality reduction is CONCOCT [56]. Here, the combined profile is constructed simply by concatenating the two vectors (*k*-mer frequency, and coverage) together while dimensionality reduction is carried out by simple unweighted PCA. All efficiency evaluators of CONCOCT were proven to grow with the number of samples; therefore, the minimal requirement for number of samples was empirically stated to be 50. CONCOCT uses a variational Bayesian approach for cluster number estimation in combination with the Gaussian mixture model (GMM) [57], which makes the binning independent of human input. Similarly to CONCOCT, COCACOLA [58] works with one feature vector, combining coverage across many samples with genomic signatures. This technique uses L_1_ distance instead of commonly used Euclidian distance to provide more reliable taxonomic binning results. Furthermore, the binning method combines advantages of soft, as well as hard clustering which eventually ensues in more robust results. COCACOLA, in addition to that, enables the incorporation of extra knowledge in the form of linkage of contigs provided by pair-end reads, and co-alignment to reference genomes into the binning method in order to enhance the binning performance.

MyCC [59] uses k-mer frequency vectors with optional addition of coverage information, which makes the method either hybrid, or composition based, in case that the coverage information is not available. The initial workflow is identical to the one used in VizBin from composition based binning section, but instead of human-augmented clustering, the affinity propagation algorithm is used for the creation of initial clusters, which are then fine-tuned by the identification of single-copy marker genes within the clusters. The universal single-copy marker genes are conserved in the majority of all sequenced bacteria and occur in exactly one copy [60] and can therefore be used as a measure of genome completeness or in case of MyCC as a useful tool for binning refinement. Although the binning method in MyCC is automatic, the affinity propagation algorithm has large memory demands and therefore, with constantly growing amount of metagenomics data, leaves space for further improvements.

Another technique working simultaneously with many samples is MetaBAT [61]. In this case, the method does not form a compact feature vector, but instead calculates probabilistic distances between pairs of sequences based on *k*-mer frequencies and abundance, and merges them into one composite distance. The probabilistic distances of tetranucleotide frequencies are computed utilizing an empirical model obtained by comparison of inter- and intraspecies distances of the known genomes, therefore, the validity of the model is verified only by the knowledge of already sequenced genomes. Even though the clustering method is fully automatic, the user is required to select one of the five predefined options regarding to the desired sensitivity and specificity. Same as MetaBAT, also MaxBin [60] works with probabilistic models. While the model based on the tetranucleotide frequencies was determined similarly using inter- and intraspecies Euclidian distances of 3181 known bacterial genomes, the difference can be found in the estimation of the model for coverage based probability distances. For a given pair of sequences, MetaBAT utilizes an area shared under normal distribution curves for quantification, whereas MaxBin adapts the Lander-Waterman model with the Poisson distribution. Clustering in MaxBin is then performed by EM algorithm. The technique uses universal single-copy marker genes for estimating the number of bins, parameter initiation and for polishing the binning output after running the EM algorithm. The original version of MaxBin was designed on single-sample data usage; however, the upgraded version, MaxBin 2.0 [62], has already allowed usage on multi-sample data, which leads to better binning results.

Software for binning of metagenomics data from several samples, such as, GroopM [63], uses primarily differential coverage of the samples accompanied by principal components of genomic signatures and by contig lengths. In this method, coverage of each sample represents one dimension in a high-dimensional space, which is then transformed by the use of unique transformation to 3D space in order to enable visualization. The binning is then performed in several steps, including two way clustering, followed by Hough partitioning on parameters formed by all, differential coverage, first principal components obtained from genomic signatures carrying at least 80% of variability, and by contig lengths. The preliminary bins are then refined by use of SOM and an optional user input.

Compared to the aforementioned methods, Differential Coverage Binning [64] uses dual information of one sample obtained by the application of two different DNA extraction methods (HP^+^, HP^−^). The doubled information regarding the coverage of each sample allows a simple visualization in 2D space. The groups in the plot are labeled according to the occurrence of essential single-copy marker genes, allowing the user to manually select a desired cluster, which is then further processed by extracting genomic signatures and the performance of PCA providing useful information in further species segregation. In the next step, pair-end reads are used for creating a network, which after visualization in Cytoscape [65], can enhance the binning results. Although the method enables advanced visualization, binning is affected purely by a user. The last method introduced in this section: MetaCluster 5.0 [66] is a tool designed for single-sample binning. MetaCluster 5.0 is able to work with short reads (75 bp), and compared to its previous version MetaCluster 4.0 [67], also deals with issue of problematic separation of low-abundant species by two-round binning. The method first separates data into three groups based on coverage, namely (i) high abundance, (ii) low abundance, and (iii) extremely low abundance sequences. The extremely low abundance sequences are filtered out and binning is then done on the two remaining groups separately. In each group, the sequences are first clustered into preliminary bins referred to as *virtual contigs* based on content of identical long *w*-mers, where *w* is high. The whole virtual contigs are then processed as single sequences and based on their *k*-mer frequencies content clustered into the final bins by automatic k-means clustering algorithm using Spearman distance. The approach from MetaCluster 5.0 has also been adapted for utilization in an annotation pipeline and is named MetaCluster-TA [68].

### 2.4 Input data

Particular groups of different taxonomy independent strategies lay different requirements on input data; while the sequence composition based strategies can work directly with sequencing reads, the classification of very short fragments is problematic due to the high variation of DNA composition patterns within a single genome. Raw sequencing reads can be therefore processed by certain tools only if their length is sufficient. In general, the length of 2000 bp is considered to be a minimum, but the longer the sequences are, the better the binning result becomes. Direct classification of raw sequencing reads is therefore possible only for third generation sequencing platforms, e.g. PacBio [69] or Oxford Nanopore [70]. The abundance based methods utilizing distribution of sequenced reads following the Lander-Waterman model, on the contrary, can work with reads as short as 75 bp, making them capable to reliably bin reads from next generation sequencing platforms, e.g. Illumina, Roche 454 or Ion Torrent [71]. The remaining abundance based techniques, as well as most of hybrid methods usually require *de novo* assembly prior to binning, as they work with coverage profiles of analyzed contigs. Although standard *de novo* assembly tools, e.g. SOAPdenovo2 [72], Celera [73], Velvet [74], etc., can provide satisfactory assembly results, preferably novel specialized assemblers for metagenomic datasets, e.g. MetaVelvet [75], IDBA-UD [76], Ray Meta [77], etc., should be used. Contigs assembled from the next generation sequencing reads can be, of course, also used as an input for composition based methods to improve their performance.

The list of all tools including requirements for input data, programming language or interface and operating system is provided in Table 1. Basic knowledge of unix based OS and scripting languages is usually required as only 3 tools are equipped with graphical interface of which the single one can be considered as a standalone application. The rest of tools are rather packages using only command line interface. Several methods are only pipelines associating different algorithms by simple command line interface. Links for downloading the tools are also included in Table 1, except for 2Tbinning tool which is no longer available.

## 3 Visualization

Since taxonomy independent techniques perform clustering in unsupervised manner, the use of interactive inspection and visualization tools can be suitable for validity verification of the binning output. This way the user obtains a comprehensive outcome and, in case of GroopM, is also able to fine-tune the results of the automatic clustering methods [63]. The visualization methods use information regarding sequence composition or coverage to produce coordinates in two- or three-dimensional space to describe the given sequence. In some cases, the sequences can be visualized simply by the use of the parameters as coordinates without any further transformation. Such an example can be seen in LikelyBin, where each dimension is represented by loglikelihood of a given generated model, or in the first clustering tier of 2Tbinning, where one axis represents the GC content, while the second OFDEG of the sequence. Also the Differential Coverage Binning method uses simple coverage information of the two samples as sequence coordinates in the first visualization step.

In the majority of cases, the sequences are described by more than three parameters, disabling them from being projected into a humanly comprehensible space. Denouement is then brought by use of dimensionality reduction techniques, such as PCA, SOM, or t-distributed Stochastic Neighbor Embedding (t-SNE). Simple PCA is used on genomic signatures in the second visualization round in 2Tbinning, or in Differential Coverage Binning, in order to improve the species resolution in a selected group obtained by different visualization technique in the first round. Another method that uses simple PCA, this time on coverage-composition vectors, is CONCOCT. In CONCOCT the clusters are visualized in a 2D space by use of the first two principal components and highlighted by specific ellipses, which carry the information about the Gaussian mixtures used for the clustering. A modified version of PCA is used in CompostBin, where principle components are weighted based on sequence abundance within a sample. Such transformation allows the formation of more distinct clusters and therefore improved visualization. Another widely used dimensionality reduction technique in metagenomics is SOM. This technique has been used on both sequence composition based [32] and on abundance based data [78]. Although the technique can provide accurate results, contour definition can be a cumbersome task; moreover, the transformation lacks from quadratic time complexity, making SOM an unpleasantly time-consuming approach. Gisbrecht et al. [79] conducted a study on dimensionality reduction techniques in metagenomics. The research compared PCA with generative topographic mapping (GTM) [80], which is a probabilistic counterpart of SOM, and with t-SNE [81]. The study pointed out t-SNE as the most suitable dimensionality reduction method, but also mentioned its drawback in the form of quadratic time complexity. This issue has been solved by Laczny et al. [47], who used a modified version of t-SNE, called BH-SNE [46], reducing the time complexity to *O*(*n* log *n*). The suitability of the method for visualization reflects its broad use. Apart from the original visualization application VizBin, it has been incorporated into MyCC binning software and into IMP: a pipeline for reproducible integrated metagenomic and transcriptomic analyses [82].

Compared to previously mentioned techniques, GroopM uses an entirely unique transformation method for dimensionality reduction. The high-dimensional data in the form of differential coverage across multiple samples, where each sample forms one of N dimensions, is projected through the origin of the hyperplane X + Y + … + N = 1 into a human friendly 3D space. Similarly to the BH-SNE application on genomic signatures, this projection method also forms rather distinguished clusters. Another possibility to visualize metagenomic data is introduced as the third visualization step in Differential Coverage Binnig (after the visualization based on differential coverage and PCA of *k*-mer frequency of selected cluster) in the form of network based information obtained from paired-end reads. Such a network can further facilitate the binning process and interpretation of the metagenomic data.

## 4 Summary and outlook

Both of two main groups of taxonomy independent binning approaches, composition as well as abundance based strategies, have their own advantages and limitations. While composition based techniques usually provide clear visualization of analyzed microbiomes, they require relatively long sequences and are not reliable for complex microbial populations with low abundant communities. The second group of strategies, on the contrary, offers complementary properties. Abundance based techniques are capable to classify short reads of complex populations with many low abundant communities, without the ability to provide clear visual result. The third group of hybrid techniques combines both strategies to overcome particular drawbacks and combines some useful properties.

An important step in the classification of metagenomic datasets is a metagenome *de novo* assembly. This step precedes the actual binning and is not incorporated into the presented tools. However, the information acquired during classification can be used, not only to infer biological knowledge, but for additional reassembly of the datasets. Therefore, it can be expected that novel tools for taxonomy independent binning will be combined with specialized metagenomic assemblers into complex pipelines for metagenomic, metatranscriptomic and metabolomic analyses. One of the first efforts of such pipelines can be already found in the abovementioned IMP pipeline.

## Figures and Tables

**Table 1:** List of tool for taxonomy independent binning.

Method	Software type	Input data	Programming languages*	Interface	Operating system	Available from
SOM např.	Package	Raw reads or contigs	Perl	CLI	Linux	https://github.com/tetramerFreqs/Binning
LikelyBin	Package	Raw reads	Perl, C	CLI	Linux	http://ecotheory.biology.gatech.edu/downloads/likelybin
SCIMM	Package	Raw reads or contigs	Python	CLI	Linux	http://www.cbcb.umd.edu/software/scimm/
2Tbinning	−	−	−	−	−	No longer available
MetaWatt	Package	Assembled contigs	Java	CLI, GUI for data exploration	Linux, Mac OS	https://sourceforge.net/projects/metawatt/
VizBin	Standalone	Contigs	Java	GUI	Linux, Mac OS, Windows	https://claczny.github.io/VizBin/
AbundanceBin	Package	Raw reads	C ++	CLI	Linux	http://omics.informatics.indiana.edu/AbundanceBin/
Canopy	Package	Gene abundance profiles	C ++	CLI	Linux, Mac OS	https://bitbucket.org/HeyHo/mgs-canopy-algorithm/wiki/Home
MBBC	Package	Raw reads	Java	CLI, GUI	Linux, Windows	http://eecs.ucf.edu/~xiaoman/MBBC/MBBC.html
CompostBin	Package	Raw reads	C, Matlab	CLI	Linux	https://sites.google.com/site/souravc/compostbin
MetaCluster	Package	Raw reads (only pair-ends)	C ++	CLI	Linux	http://i.cs.hku.hk/~alse/MetaCluster/index.html
Dif. Cov. Bin.	Pipeline	Raw reads	R	CLI	Linux	https://github.com/MadsAlbertsen/multi-metagenome
CONCOCT	Package	Contigs + BAM	Python	CLI	Linux, Mac OS	https://github.com/BinPro/CONCOCT
MaxBin	Package	Contigs + (reads or abundance file)	Perl	CLI	Linux, Mac OS	https://sourceforge.net/projects/maxbin/
GroopM	Package	Contigs + BAM	Python	CLI	Linux	http://ecogenomics.github.io/GroopM/
MetaBAT	Pipeline	Contigs + BAM	C ++	CLI	Linux	https://bitbucket.org/berkeleylab/metabat
COCACOLA	Pipeline	Contigs + raw reads	Matlab	CLI	Linux	https://github.com/younglululu/COCACOLA
MyCC	Package	Contigs + BAM*optional	Python	CLI	Linux	https://sourceforge.net/projects/sb2nhri/files/MyCC/
